# Draft Genome Sequence of *Burkholderia* sp. Strain WAC0059, a Bacterium Isolated from the Medicinal Fungus *Antrodia cinnamomea*

**DOI:** 10.1128/genomeA.00027-18

**Published:** 2018-02-08

**Authors:** Shu-Ting Cho, Chia-Ling Chen, Yin Yang, Ting-Fang Wang, Chih-Horng Kuo

**Affiliations:** a Institute of Plant and Microbial Biology, Academia Sinica, Taipei, Taiwan; b Institute of Molecular Biology, Academia Sinica, Taipei, Taiwan; c The HIMA Foundation, Taipei, Taiwan

## Abstract

*Burkholderia* sp. strain WAC0059 was isolated from a fruiting body of the medicinal fungus *Antrodia cinnamomea* collected in Taiwan. Here, we report the draft genome sequence of this bacterium to facilitate the investigation of its biology.

## GENOME ANNOUNCEMENT

The fungal species *Antrodia cinnamomea* (1), synonyms *Antrodia camphorata* (2) and *Taiwanofungus camphoratus* (3), is a popular medicinal mushroom in Taiwan that is noted for its protective effects against liver injury (4). The bacterial strain *Burkholderia* sp. WAC0059 was isolated from the fruiting body of an *A. cinnamomea* specimen wild-collected by C.-L.C. and T.-F.W. in 2013. To facilitate future investigation into the biology of this bacterium, and of its possible interactions with the fungal host, we report here a draft genome assembly of this bacterium.

The procedures for whole-genome shotgun sequencing and genome assembly were based on those described in our previous studies (5–7). Briefly, we utilized the Illumina MiSeq platform to obtain 301-bp sequencing reads from one paired-end library with approximately 1,273-fold coverage. The initial *de novo* assembly was performed using Velvet version 1.2.10 (8). Subsequently, an iterative process was used to improve the assembly. For each iteration, all raw reads were mapped to the assembly using the Burrows-Wheeler Alignment (BWA) tool version 0.7.12 (9), programmatically checked using the MPILEUP program in the SAMtools package version 1.2 (10), and visually inspected using Integrative Genomics Viewer (IGV) version 2.3.57 (11). Regions that involved possible misassembly were removed, and polymorphic sites were corrected based on the mapped reads. The annotation was performed using the Prokaryotic Genome Annotation Pipeline (PGAP) provided by the National Center for Biotechnology Information (NCBI) (12).

The first version of this draft genome contains 61 contigs with a combined size of 5,349,796 bp; the average G+C content is 66.8%. The annotation includes 4 rRNA genes, 49 tRNA genes, and 4,575 protein-coding genes.

### Accession number(s).

This whole genome shotgun project has been deposited at DDBJ/EMBL/GenBank under the accession number PNEO00000000. The version described in this paper is the first version, PNEO01000000.

## References

[1] Chang TT, Chou WN 1995 *Antrodia cinnamomea* sp. nov. on *Cinnamomum kanehirai* in Taiwan. Mycol Res 99:756–758. doi:10.1016/S0953-7562(09)80541-8.

[2] Wu S-H, Ryvarden L, Chang T-T 1997 *Antrodia camphorata* (“niu-chang-chih”), new combination of a medicinal fungus in Taiwan. Bot Bull Acad Sin 38:273–275.

[3] Wu S-H, Yu Z-H, Dai Y-C, Chen C-T, Su C-H, Chen L-C, Hsu W-C, Hwang G-Y 2004 *Taiwanofungus*, a polypore new genus. Fungal Sci 19:109–116.

[4] Liu YW, Lu KH, Ho CT, Sheen LY 2012 Protective effects of *Antrodia cinnamomea* against liver injury. J Tradit Complement Med 2:284–294. doi:10.1016/S2225-4110(16)30114-6. 24716143 PMC3942906

[5] Lo WS, Chen LL, Chung WC, Gasparich GE, Kuo CH 2013 Comparative genome analysis of *Spiroplasma melliferum* IPMB4A, a honeybee-associated bacterium. BMC Genomics 14:22. doi:10.1186/1471-2164-14-22. 23324436 PMC3563533

[6] Ku C, Lo WS, Chen LL, Kuo CH 2013 Complete genomes of two dipteran-associated spiroplasmas provided insights into the origin, dynamics, and impacts of viral invasion in *Spiroplasma*. Genome Biol Evol 5:1151–1164. doi:10.1093/gbe/evt084. 23711669 PMC3698928

[7] Lo WS, Ku C, Chen LL, Chang TH, Kuo CH 2013 Comparison of metabolic capacities and inference of gene content evolution in mosquito-associated *Spiroplasma diminutum* and *S*. *taiwanense*. Genome Biol Evol 5:1512–1523. doi:10.1093/gbe/evt108. 23873917 PMC3762197

[8] Zerbino DR, Birney E 2008 Velvet: algorithms for *de novo* short read assembly using de Bruijn graphs. Genome Res 18:821–829. doi:10.1101/gr.074492.107. 18349386 PMC2336801

[9] Li H, Durbin R 2009 Fast and accurate short read alignment with Burrows–Wheeler transform. Bioinformatics 25:1754–1760. doi:10.1093/bioinformatics/btp324. 19451168 PMC2705234

[10] Li H, Handsaker B, Wysoker A, Fennell T, Ruan J, Homer N, Marth G, Abecasis G, Durbin R; 1000 Genome Project Data Processing Subgroup 2009 The Sequence Alignment/Map format and SAMtools. Bioinformatics 25:2078–2079. doi:10.1093/bioinformatics/btp352. 19505943 PMC2723002

[11] Robinson JT, Thorvaldsdóttir H, Winckler W, Guttman M, Lander ES, Getz G, Mesirov JP 2011 Integrative genomics viewer. Nat Biotechnol 29:24–26. doi:10.1038/nbt.1754. 21221095 PMC3346182

[12] Tatusova T, DiCuccio M, Badretdin A, Chetvernin V, Nawrocki EP, Zaslavsky L, Lomsadze A, Pruitt KD, Borodovsky M, Ostell J 2016 NCBI Prokaryotic Genome Annotation Pipeline. Nucleic Acids Res 44:6614–6624. doi:10.1093/nar/gkw569. 27342282 PMC5001611

